# Variation in Heterozygosity Predicts Variation in Human Substitution Rates between Populations, Individuals and Genomic Regions

**DOI:** 10.1371/journal.pone.0063048

**Published:** 2013-04-30

**Authors:** William Amos

**Affiliations:** Department of Zoology, Cambridge University, Cambridge, Cambridgeshire, United Kingdom; The Scripps Research Institute, United States of America

## Abstract

The “heterozygote instability” (HI) hypothesis suggests that gene conversion events focused on heterozygous sites during meiosis locally increase the mutation rate, but this hypothesis remains largely untested. As humans left Africa they lost variability, which, if HI operates, should have reduced the mutation rate in non-Africans. Relative substitution rates were quantified in diverse humans using aligned whole genome sequences from the 1,000 genomes project. Substitution rate is consistently greater in Africans than in non-Africans, but only in diploid regions of the genome, consistent with a role for heterozygosity. Analysing the same data partitioned into a series of non-overlapping 2 Mb windows reveals a strong, non-linear correlation between the amount of heterozygosity lost “out of Africa” and the difference in substitution rate between Africans and non-Africans. Putative recent mutations, derived variants that occur only once among the 80 human chromosomes sampled, occur preferentially at the centre of 2 Kb windows that have elevated heterozygosity compared both with the same region in a closely related population and with an immediately adjacent region in the same population. More than half of all substitutions appear attributable to variation in heterozygosity. This observation provides strong support for HI with implications for many branches of evolutionary biology.

## Introduction

Mutations provide the clay on which evolution operates and understanding where and when they occur is critical to many branches of evolutionary biology. Recent studies reveal many sources of heterogeneity including higher mutation rates in some families compared with others [1], near heterozygous microdeletions [2], near mononucleotide tracts and on the lagging strand at replication origins [3]. Mutation rate also differs between related species, being lower in humans compared with other great apes [4] and in larger compared with smaller mammals [5]. Mutations also appear clustered [6], [7], suggesting either mutation hotspots [8], [9] or a tendency for one mutation locally to promote further events [10], [11], the latter supported also by the tendency for adjacent mutations to occur on the same strand [12].

One mechanism that might contribute to these heterogeneities in diploid organisms is ‘heterozygote instability’ (HI), a suggestion that mutation rate increases at and near heterozygous sites where the two homologous chromosomes differ in sequence [13]. Direct empirical support for HI comes from studies in yeast where, during synapsis, extensive regions of heteroduplex DNA are formed in which heterozygous sites appear as mismatches [14], [15]. Such mismatches are recognised by mismatch repair enzymes and these initiate gene conversion-like events [16], where the extra round of DNA replication might be expected to provide opportunities for additional mutations [10], [13]. At the population level, HI is implicated by the way human microsatellite mutation rate increases with population size and hence with heterozygosity [17], [18].

To explore the possibility that HI locally increases mutation rates, I exploited the well-documented loss of all forms of diversity that occurred when humans migrated out of Africa. Since the reduction in diversity outside Africa is found equally across SNPs, microsatellites, neutral morphological variation and even commensal gut bacteria diversity [19]–[22] there seems no question that the primary causative factor is one or more population bottleneck(s) [19], [23], [24]. Under the HI hypothesis, this demographically-induced reduction in heterozygosity should create a parallel reduction in mutation rate such that Africans have diverged more than non-Africans from their common ancestor.

## Methods

### Data

I analysed publicly released data from the thousand genomes project [25]. I downloaded all available human (Hg19) – chimpanzee (PanTro2) pairwise alignments from the UCSC Genome Browser using the Galaxy toolbox (galaxy.psu.edu). Short alignments (<1000 bases) were excluded because many are unreliable due to the presence of repetitive sequences. For a range of human genomes from diverse populations I downloaded the ‘diversity’ samples from ‘Complete Genomics’ (www.completegenomics.com) [26]. This sample set includes at least four individuals from each of 10 diverse populations. To obtain equal contributions from each population I took all available individuals from populations represented by four samples (most) and selected four random individuals from each of the remaining populations that have more than four individuals = 40 individuals total (LWK, Lahuya from Kenya; codes = 21732, 21733, 21737, 21767: MKK, Maasai from Kenya; 19017, 19020, 19025, 19026: YRI, Yoruban from Nigeria; 18502, 18504, 18508, 18517: TSI, Toscans from Italy; 20502, 20509, 20510, 20511: CHB, Han Chinese from Beijing; 18526, 18537, 18555, 18558: JPT, Japanese from Tokyo; 18940, 18942, 18947, 18956: UTA, European descent from Utah; 06994, 06984, 07357, 10851: ASW, African ancestry from USA; 19700, 19701, 19703, 19704: GUJ, Gujurati Indians from USA; 20850, 20845, 20846, 20847: MXL, Mexican ancestry from USA; 19669,19648, 19649, 19670). I chose not to use all available samples because in some of the downstream analyses I use pairwise comparisons where, to avoid bias, each population must contribute equally. From the perspective of statistical power, four individuals may seem rather small. However, recombination ensures that each individual contains a patchwork of fragments derived from many different lineages within their parent population and, with a sample size of 2 billion bases, even a single individual should be considered a reasonably representative and large sample of the genetic characteristics of the population from which it was drawn.

In some cases, individuals were sampled under circumstances where the possibility of mixed ancestry is enhanced. Population samples were considered potentially admixed if their country of sampling differed from their population origin (i.e. UTA, ASW, GUJ, MXL). To eliminate possible biases due to missing data, only bases called with high confidence in all individuals were accepted. Since adjacent polymorphic bases may indicate poor quality alignments, only polymorphisms flanked on both sides by bases that were monomorphic across the chimpanzee and all humans were accepted. Downloaded sequence data were extracted, aligned and analysed using custom scripts written in C++ (available on request).

### Calculating Substitution Rates

Classical theory states that the (diploid) per generation substitution rate, *k*, depends only on the effective population size, *N*, and the mutation rate, µ:
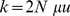
where *u* is the probability of single mutant reaching fixation (see [27], page 46). Since *u* = 1/2 N, over the long term the above equation simplifies to *k* = µ. Over the short term, population size fluctuation can create imbalances between the number of mutations entering a population each generation (2 Nµ) and the probability that any one mutant allele becomes fixed. To circumvent such demographic dependencies I therefore used a variant of the relative rate test [28] applied to individual aligned haploid bases. Consider three orthologous bases, one from the chimpanzee (C) and one from each of two different humans (H1, H2). These bases can be placed on a simple ‘tree’ that splits twice, once between humans and chimpanzees and once within humans. Since the three bases are identical in ∼99% of cases and double mutations are extremely rare, whever one base differs from the other two, the singleton base most likely represents a substitution in the lineage it represents. For example, if C = A, H1 = A and H2 = G, an A->G substitution is inferred in the lineage leading to H2.

If generation length and mutation rate are constant, the probability, S, of a substitution occurring at one site in one lineage is given by:
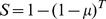
where T is the branch length in generations. There are two important implications. First, there is no population size term, so demography has no influence. Second, the expectation for the *difference* in S between two human lineages can only differ from zero if either µ or T differs between the two lineages. This is true despite the fact that recombination and demographic processes cause T to vary along a chromosome because, for any one base, the gene tree is always symmetrical: with only two branches, geologic time since the most recent common ancestor must be the same on both branches. From hereon, unless otherwise stated, I use substitution rate to refer to the proportion unambiguously aligned bases in a given lineage in which a substitution is inferred. Note, this definition is a pairwise property and for any one individual will vary depending on the other individual being compared.

Substitution rates were calcualted from pairwise comparisons between all 40 human samples. With one haploid chimpanzee reference sequence, in any given comparison four possible chimpanzee-human1-human2 trios can be made. i.e. both bases in human1 against both bases in human2. Since phase is not known, all four comparisons are made and the resulting number divided by four to give the equivalent haploid number. The relative substitution rate (RSR) for individual *i* compared against individual *j* (RSR*_ij_*) is calculated as (M_i_–M_j_)/N, where M_i_ and M_j_ are the total numbers of substitutions inferred in the lineages leading to *i* and *j* respectively since their common ancestor and N is the number of qualifying bases. N is effectively the same for all individuals because only bases called in all individuals are included and the number of non-qualifiying bases where all three bases differ is only 0.1% of qualifying bases. For any given individual *i*, mean RSR is calculated as the average RSR*_ij_* across all non-self comparisons. In other words, mean RSR is the average tendency for an indivdual to carry more or fewer derived mutations relative to all 39 other humans in the dataset.

### Regional Variation in Substitution Rate and Heterozygosity across the Genome

To explore the extent to which heterozygosity and substitution rates vary across the genome, sequence data were partitioned into non-overlapping blocks of 2,000,000 qualifying bases in the chimpanzee-HG19 alignment, typically equivalent to 2.4 Mb on a chromosome map, but sometimes much larger in telomeric and centromeric regions where many bases are not scored/aligned. Since the expected signal is likely to be weak relative to the number of bases sampled in each window, it is important to minimise unnecessary sources of statistical noise. Admixed individuals may contain a patchwork of genomic regions from more than one geographic origin, a pattern that could contribute appreciably to the error variance. Consequently, for maximum purity of signal I used only individuals thought not to be appreciably admixed: LWK, MKK and YRI for Africa and TOS, CHB and JPT for non-Africans. Differences in African – non-African RSR and heterozygosity were obtained by averaging across the 12 individuals in each group. Essentially identical results are obtained if RSR is replaced by the more straightforward measure of human-chimpanzee divergence, quanitifed as the proportion of all qualifying bases that differ with no adjustment for transitions versus transversions.

## Results

### Substitution Rate Variation between Individuals, Populations and Chromosomes

**Figure 1:**
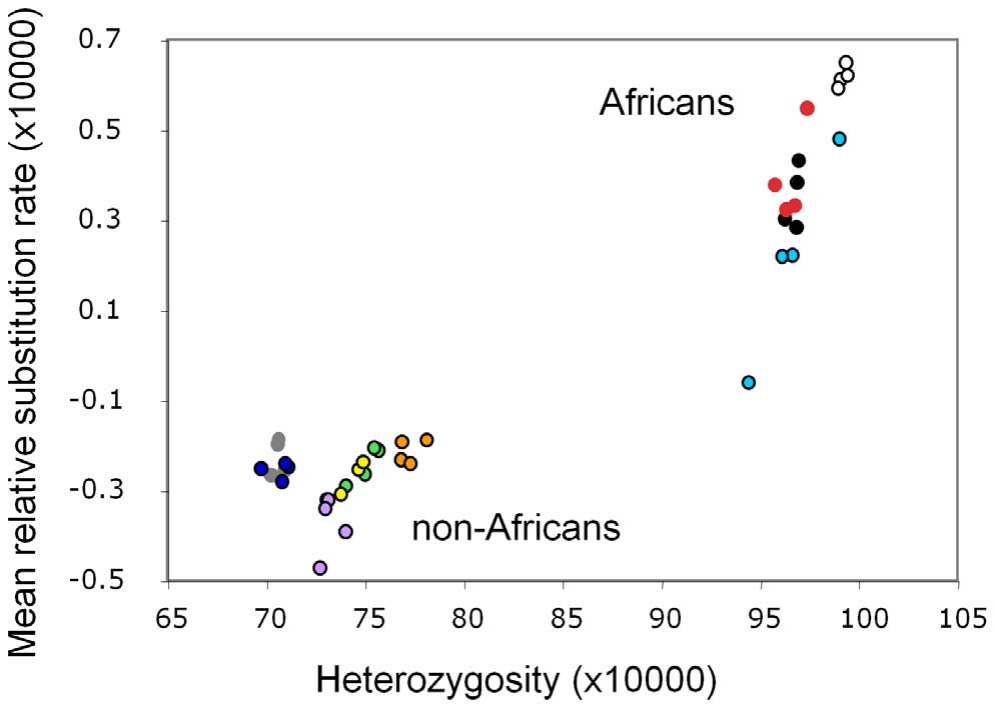
Relationship between substitution rate and heterozygosity among 40 humans from 10 populations. Populations are: LWK (red), MKK (black), YRI (white), TSI (green), CHB (grey), JPT (dark blue), UTA (yellow), ASW (light blue), GUJ (orange), MXL (purple). Abbreviations are given in methods. Mean relative substitution rate is calculated for each individual relative to all others, negative scores indicating lower than average and positive indicating above average, and expressed as substitutions per qualifying base. Heterozygosity is calculated as the proportion of heterozygous sites among all qualifying bases. Both axes are scaled for clarity. Standard errors for each data point are nominally of the order of 0.035, making them too small to show clearly as error bars.

Figure 1 depicts a plot of the average tendency for any given human to carry more or fewer substitutions relative to all other humans, the mean relative substitution rate (RSR, see methods), against genome-wide heterozygosity, calculated as the number of heterozygous bases in that individual divided by the total number of qualifying bases considered. Two non-overlapping clusters are seen: all Africans have both higher heterozygosity and higher mean RSR compared with all non-Africans. Moreover, all 10 population groups individually exhibit a positive correlation between genomewide heterozygosity and mean RSR among their four component individuals (mean correlation coefficient, r = 0.57 significantly >0: t = 5.01, 9d.f., P = 0.0007, two-tailed). The widest range of values and the strongest correlation (r = 0.99) both relate to the African-American group, ASW. This likely reflects varying levels of non-African admixture causing unusually high levels of variation in both heterozygosity and substitution rate. Variation in heterozygosity is less marked because, while substitution rate reduces linearly in proportion to non-African admixture, heterozygosity reduces approximately in proportion admixture squared; i.e. the probability of being homozygous non-African (dissimilarity between two African chromosomes will be similar to that between one African and one non-African chromosome).

**Figure 2:**
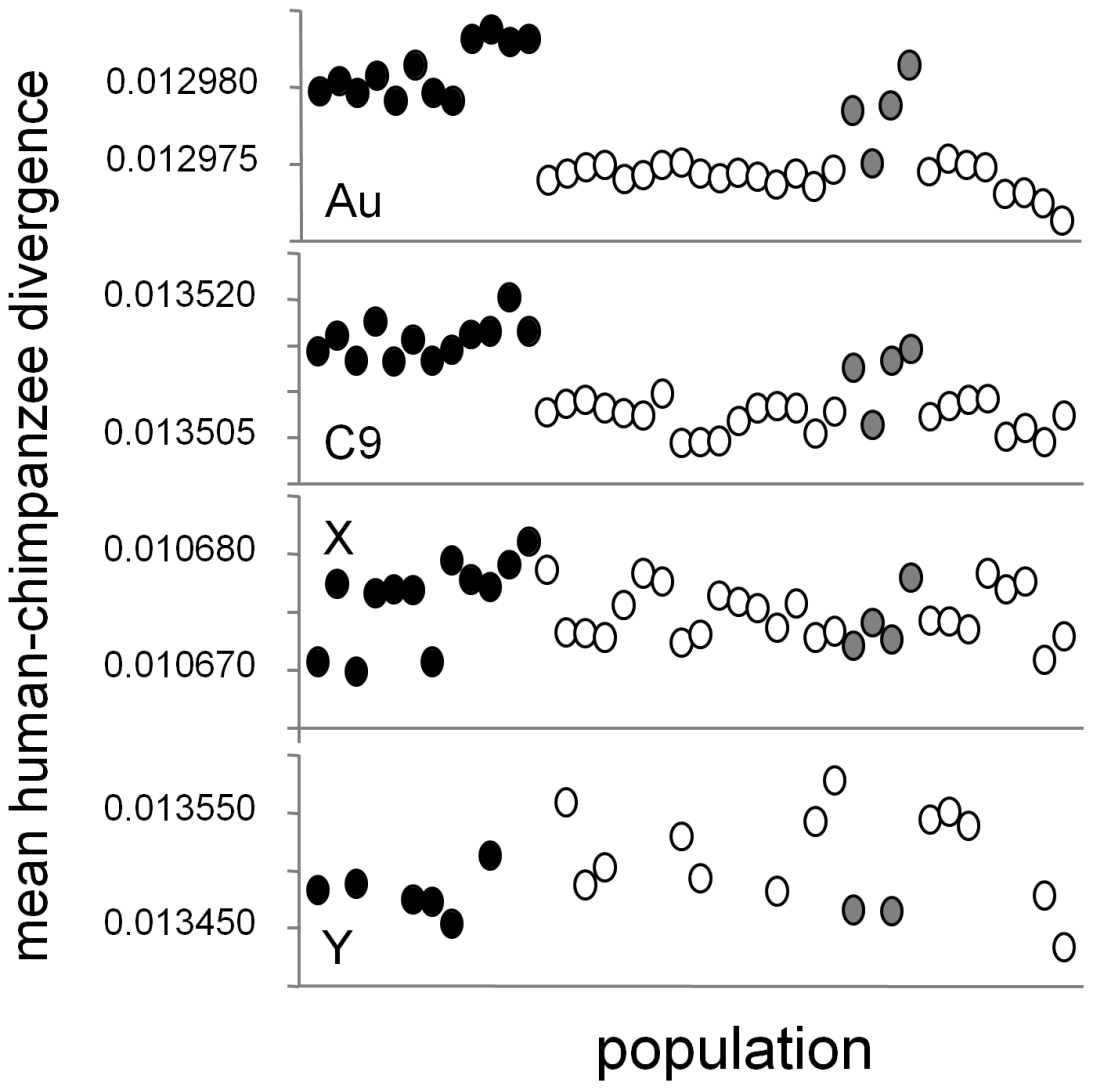
Genetic divergence of 40 humans from the chimpanzee reference sequence according to genomic region. Divergence rates are quantified as the proportion of qualifying bases that differ with no adjustment for differences in rate of transition and transversions. Populations are: Africans sampled in Africa (black, ordered from left LWK, MKK, YRI), Africans sampled in America (ASW, grey) and non-Africans (open circles, four of each ordered from left TOS, CHB, JPT, UTA, GUJ, MXL). Panels are: all autosomes (Au); a randomly-selected medium sized autosome, chromosome 9 to compare with the X (C9); the X chromosome (X); the Y chromosome (Y, only half the samples are males). All autosomes yield very similar patterns with all Africans showing higher divergence than all non-Africans.

If heterozygosity modulates mutation rate, its effects should be absent/reduced in haploid/semi-haploid regions of the genome respectively. The available population samples differ in their numbers of males and females, potentially making RSR biased. I therefore tested this prediction using a less sensitive measure of substitution rate, the human-chimpanzee divergence, estimated as the average probability that a given base differs between the human chromosomes and the chimpanzee reference sequence. As elsewhere, only bases called in all individuals are included. Africans exhibit greater divergence than non-Africans on the autosomes, but in line with the prediction, reduced and zero differences are seen on the X and Y chromosomes respectively (Figure 2). Interestingly, human-chimpanzee divergence is significantly lower on the X compared with the autosomes (0.0101 compared with 0.032, the mean of 22 individual estimates for the autosomes: t = 18.05, 21d.f., P = 2.9×10^−14^), consistent with a long-term lower mutation rate on this semi-diploid chromosome.

### Regional Variation in Substitution Rate

**Figure 3:**
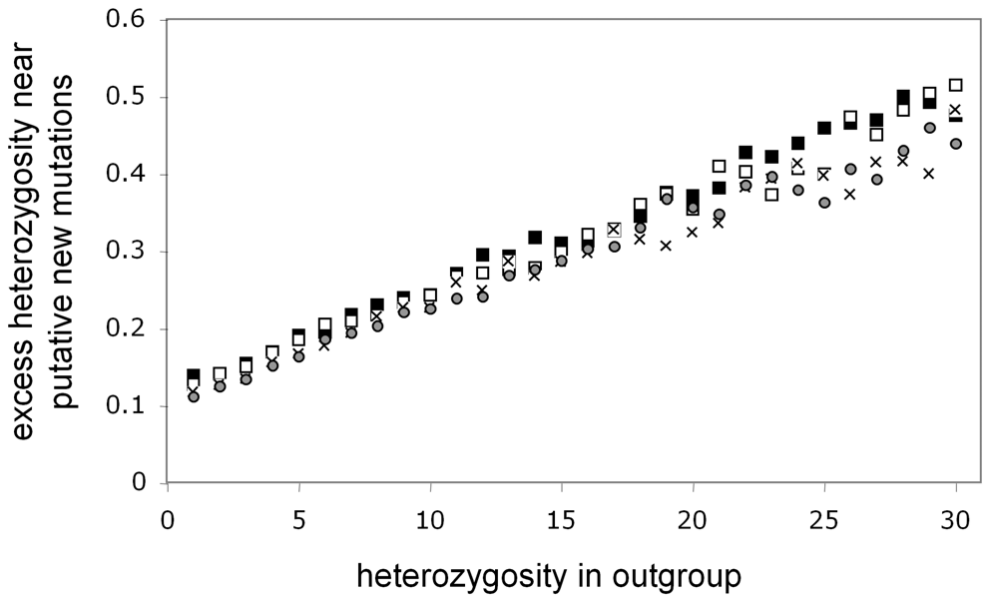
Degree of ‘out of Africa’ loss of heterozygosity predicts African – non-African substitution rate difference across 1272 genomic regions. The genome was divided into ∼2 Mb non-overlapping windows and, within each, values derived for the average heterozygosity and average substitution rate in 12 non-admixed Africans (LWK, MKK, YRI) and 12 non-admixed non-Africans (TSI, CHB, JPT) yielding paired difference values. The raw data exhibit considerable scatter. For clarity, data were binned by size of heterozygosity difference and both axes were rescaled for clarity. Three data points are omitted where partial windows at the end of a chromosome yielded appreciably fewer qualifying bases. The third order polynomial fitted to the means is highly significant (r^2^ = 0.993, t = 39.54, 11 d.f., P = 3.3×10^−13^) as are linear and polynomial regressions fitted to the raw data (see text). Changes in heterozygosity are dominated by the ‘out of Africa’ bottleneck, modulated by natural selection, hence are almost invariable positive (heterozygosity greater in Africa).

Loss of genetic diversity ‘out of Africa’ is by no means uniform across the genome, but instead was modulated by selection, acting in some cases to reduce and elsewhere to accelerate loss [29]. The HI hypothesis predicts that such variation should create parallel variation in the magnitiude of the African – non-African substitution rate difference. I therefore reanalysed the data as a series of non-overlapping 2 Mb windows, revealing a highly significant positive correlation between diversity lost and substitution rate difference (Figure 3, correlation calculated on unbinned data with significance determined as a t-approximation, t_[1286]_ = 6.3, P = 4.8×10^−10^). Fitting a third order polynomial, as suggested by the plot appreciably strengthens the relationship (t-approximation: t_[1286]_ = 7.1, P = 2.2×10^−12^). Since neighbouring windows are somewhat non-independent due to linkage disequilibrium, I also tested for consistency of effect across the genome by calculating separate (linear) regressions for the same variables in all windows present on each of the 22 autosomes. For all but the smallest chromosome, 22, the slopes of the regressions are positive. This represents a significant deviation from null hypothesis of 1∶1 positive to negative slopes (**Χ** ^2^ [_1_] = 14.7, P = 0.00012) and mean r is significantly greater than zero (mean r = 0.187±0.031 s.e., t = 6.1, 21 d.f., P<10^−6^).

### Do New Mutations Occur Preferentially in Regions of High Heterozygostiy?

**Figure 4:**
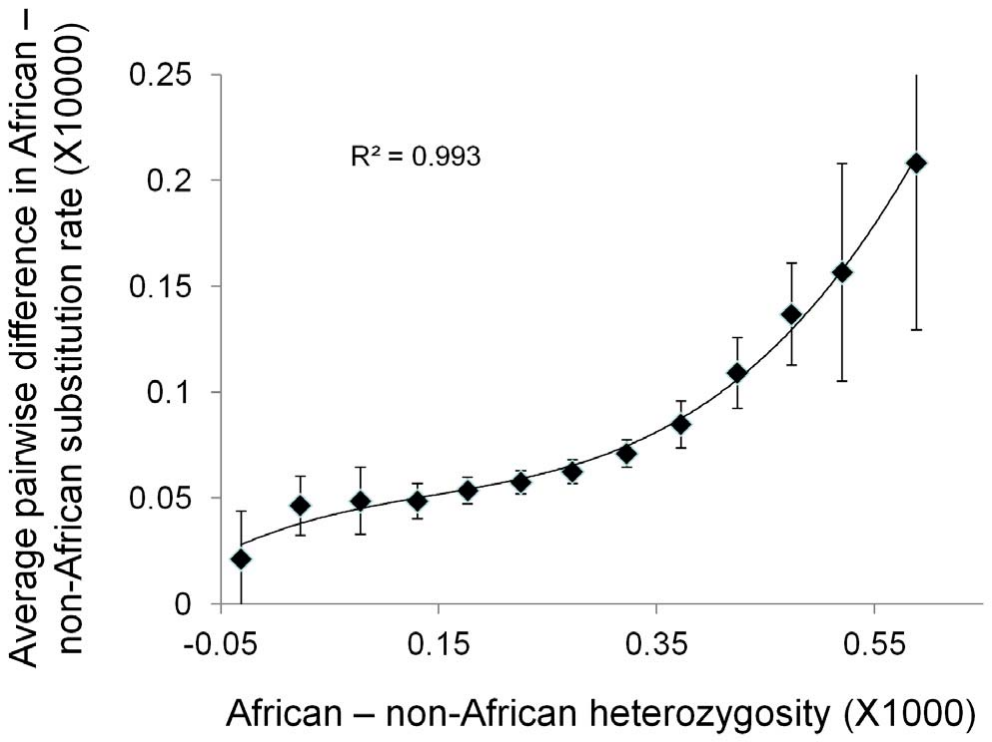
Average difference in heterozygosity between closely related populations in regions surrounding putative new mutations (PNMs). PNMs are defined as derived variants that occur only once among 80 human chomosomes studied. The x-axis is heterozygosity in a less related outgroup population, quantified as total number of heterozyous sites in the 2 Kb window summed over all four individuals, while the y-axis is the difference in heterozygosity between the population in which the PNM was inferred and a closely related sister population, measured as average excess sites per individual. Reciprocal comparisons were conducted between two pairs of sister populations: Uta (open squares) and Tos (black squares) in Europe and Chb (grey crosses) and Jpt (grey circles) in east Asia. Outgroups were Jpt and Tos four Europe and East Asia respectively. In all cases, heterozygosity in the population in which the PNM was inferred is on average greater than in the control populaiton, this difference rising as heterozygosity increases in the outgroup. Results presented are weighted linearly by proximity to the putative new mutation, but an unweighted analysis yields essentially identical results.

A direct test of HI would count *de novo* mutations in pedigrees, but a reasonable sample size of mutations across a range of individuals, populations and genomic contexts would require a prodigious sequencing effort. As a compromise, I examined the distribution of putative recent mutations (PRMs), defined as derived (differing from the chimpanzee reference) variant bases that occur only once in the 80 human chromosomes sampled. Around each PRM found, heterozygosity in that population is assessed in a centred, 2 Kb window. This is then compared both with the same window in a closely related population and with a randomly selected window in the same population, chosen to lie between 1 Kb and 2 Kb away. To avoid bias, the individual in which the PRM was found is always excluded from calculations of heterozygosity. In four reciprocal comparisons between two pairs of closely related populations (Europe = TOS and UTA; East Asia = CHB and JPT) average heterozygosity is consistently higher in the population in which the PRM was inferred (Figure 4). Using the same four populations, heterozygosity around the PRM is also consistently higher than in an adjacent control region 1–2 Kb distant (mean focal – control differences: Tos 0.006±0.002 s.e., Uta 0.008±0.002 s.e., Jpt 0.004±0.002 s.e., Chb 0.005±0.002 s.e.). All comparisons are significant at P<0.01 except the Japanese, which is borderline (P = 0.06). A slightly more distant control window at 3–4 Kb distant yields substantially more significant differences (all P<2×10^−4^), suggesting a highly localised pattern with a sphere of influence extending appreciably <5 Kb.

### Calculating the Effect Size

Since even negligible trends could become highly significant when analysed in a large dataset such as a complete genome, I attempted to estimate the likely HI effect size by comparing substitution rates in Africans and non-Africans. Yorubans (YRI) typically carry ∼9,000 more substitutions per haploid genome and a third more heterozygous sites than a typical non-African. If the excess substitutions are all attributed to mutations at or near heterozygous sites, the total heterozygosity-attributable substitution rate would be 4×9,000 = 36,000 events, or 18 per generation since the African – non-African split approximately 50,000 years/2,000 generations ago [30]. With 2.04×10^9^ aligned bases considered in the current analysis, this equates to a per base per generation substitution rate of 9×10^−9^, almost half the genome-wide mutation rate, 2×10^−8^, estimated from pedigrees [31]. Moreover, this is likely to be an under-estimate because heterozygosity is lost slowly following population decline.

## Discussion

Here I test the hypothesis that ‘repair’ of heteroduplex DNA during synapsis will cause an increase in mutation rate at and around heterozygous sites, referred to as ‘heterozygote instability’ or HI. I exploit the well-established loss of variability as humans left Africa to ask whether there has been a parallel reduction in substitution rate. I find that, both genome-wide and regionally across the genome, the apparent substitution rate has been reduced and that the extent of the reduction correlates with the amount of variability lost. Moreover, this pattern seems restricted to diploid regions of the genome where the pairing of homologues allows heterozygous sites to be recognised.

A critical aspect of my analysis is the way in which demographic effects are removed from the calculation of substitution rates by effectively estimating the mean probability that any given base in a person’s genome is derived rather than ancestral with respect to the chimpanzee. In this way I avoid possible problems of circularity where population bottlenecks impact both heterozygosity and substitution rate in parallel. That effective separation of the two measures has been achieved can be seen by looking at the African American samples, which exhibit varying levels of non-African admixture. These individuals have rather similar heterozygosities but widely differing substitution rates, showing clearly that heterozygosity and substitution rates can vary independently. An alternative, verbal argument is that a bottleneck acts blindly to reduce variability, and is therefore as likely to cause any given derived variant to increase as to decrease in frequency. Across a large sample of bases, the expectation for the net change in frequency of derived variants following a bottleneck must therefore be zero: substitution rate is unaffected.

A tendency for Africans to have diverged more from chimpanzees than non-Africans is unexpeced under classical theory. Since the substitution rate I measure depends only on generation number and mutation rate, one or both of these must have changed as we left Africa. Two models seem possible, local effects that vary across the genome due, for example, to natural selection, and genome-wide effects arising from a mutator allele impacting mutation rate or demographic influences that alter generation time. Certainly, generation length estimates vary considerably between the sexes and between populations across the globe [32]. However, in the absense of HI both genome-wide and local models are difficult to reconcile with different aspects of the current analysis.

Most plausible local effects models are based on natural selection. At any one genomic location natural selection might act to favour either greater longevity, thereby lengthening generation time, or early reproduction, thereby shortening generation length. However, there is no clear reason why such effects should be distributed across all autosomes in a way such that they always, or predominantly, favour shorter generation times in Africa. There is also no good reason why a pattern caused on the autosomes by variation in generation length between populations should fail to generate similar patterns on the sex chromosomes. Natural selection also struggles in two ways to explain the correlation between heterozyosity lost ‘out of Africa’ and difference in African – non-African substitution rate. First, a simple model of selection acting to change both heterozygosity and substitution rate would likely generate a linear relationship in contrast to the observed correlation. which is strongly non-linear. Second, just as substitution rate (as I calculate it) is not impacted by demography, neither will it be impacted by hitch-hiking in the vicinity of where selection is operating: unless a derived variant is the actual focus of selection, hitch-hiking is as likely to drive that variant up in frequency as it is to drive it down. Directly selected variants do have the potential to alter the substitution rate but these represent such a small proportion of all variants that they will contribute negligibly to the overall pattern.

Most genome-wide mechanisms such as a shorter generation time in Africa also appear to be inadequate. First, such mechanisms will generally act across all chromosomes, not just/mainly the autosomes, so in general fail to explain the lack of difference seen particularly on the haploid Y chromosome. Second, genome-wide processes would not drive the correlation between local heterozygosity difference and local substitution rate difference. Third, genome-wide models predict that putative recent mutations will be distributed randomly across the genome and not necessarily concentrated in regions with elevated heterozygosity. A similar argument can be made to a lesser extent for local effect models. Finally, we must not exclude the possibility of human error: a genomewide pattern might arise through a programming error in which the calculation of heterozygosity and substitution rates are confounded. However this seems unlikely because it would tend to generate a constant, linear relationship between the two quantities, and is therefore at odds variously with the non-linear relationship in Figure 3, the unusually steep slope seen in African Americans in Figure 1 and with the autosome – sex chromsome difference.

The only model I can conceive that is consistent with all the above findings is the hypothesis I set out to test, specifically that heterozygosity increases local mutation rate. HI has the unusual property not carried by other candidate mechanisms of being able to generate both broad brush, genome-wide patterns, but also to modulate the size of effect seen in response to factors such as ploidy or where selection has acted to reduce or accelarate the loss of diversity [29]. In particular, HI naturally accommodates the difference between autosomes and the sex chromosomes, both temporal and regional finescale differences in where new mutations occur and the non-linear correlation between difference in African – non-African substition rate and heterozygosity, where HI might plausibly cause an accelarating process.

The implications of a mutation rate that increases with increasing heterozygosity are wide-ranging, particularly if the effect size is a large as it seems. When population size is constant, smaller populations will experience lower mutation rates than related larger populations. Interestingly, two of the lowest reported microsatellite mutation rates are both based on mutation accumulation in inbred lines, one in *Drosophila* [33] and one in *Dictyostelium* [34]. For humans, HI implies a reduction in mutation rate as we left Africa with the counter-intuitive result that non-Africans will appear more closely related than Africans to other hominid lineages such as Neanderthals, a trend that has been observed and used as evidence of introgression [35]. Within a species, HI would be expected to cause regions under balancing and purifying selection respectively to exhibit enhanced and reduced mutation rates. Such a pattern could be seen as adaptive, since mutations will be directed more towards regions where polymorphism is tolerated/beneficial and away from more conserved regions. More generally, HI would add evolutionary momentum to demographically induced changes in heterozygosity, prolonging the impact of bottlenecks and exaggerating diversity gained through hybridisation. Of concern is the fact that the existence of HI would undermine the validity of classical and extremely widely used equalities relating heterozygosity to mutation rate and effective population size. Classical equations including the term Neµ will need to be revisited. Further research is needed to establish the existence and magnitude of these possible effects.

Although HI implies some rethinking of models of how genetic variability is gained, there is also the potential to develop new tools that exploit the resulting patterns. As seen above, the apparent substitution rate changes much more slowly than observed heterozygosity, and therefore can potentially be used to infer the demographic history of the genome, both in terms of regions which have experienced long-term balancing or purifying selection, or introgression between populations that differ in long-term effective population size. Phenomena like mutation hotspots might also be seen in a different light, as should variation in recombination rate, since both are likely to some extent to be exaggerated or even caused by HI: the gene conversion-like events attracted by heterozygous sites likely in some cases to be resolved by recombination.

In conclusion, whole genome comparisons reveal surprising differences in substitution rates between human populations that correlate positively with heterozygosity, consistent with the HI hypothesis. HI could help to explain a number of previously challenging observations, including the lower mutation rate in larger mammals, these tending to live in smaller populations, and the way individuals in hybrid zones tend to carry unexpectedly many novel mutations [36], [37]. The hominoid slowdown could be explained either by a reduction in population size, perhaps due to increasing sociality, or through repeated selective sweeps during the evolution of bipedality, language and increased brain size. HI should provide a fertile area for future research.
