# Correction: Variation in Heterozygosity Predicts Variation in Human Substitution Rates between Populations, Individuals and Genomic Regions

**DOI:** 10.1371/annotation/35be1800-1e9f-4b76-8b6a-660998434b13

**Published:** 2013-06-27

**Authors:** William Amos

Figures 3 and 4 were incorrectly switched. 

The image appearing as Figure 3 belongs with the label and legend for Figure 4, and the image appearing as Figure 4 belongs with the label and legend for Figure 3. The labels and legends for the two figures are in correct order.

